# Effect of Process Parameters on Weld Quality in Vortex- Friction Stir Welding of 6061-T6 Aluminum Alloy

**DOI:** 10.3390/ma16020873

**Published:** 2023-01-16

**Authors:** Xiaochao Liu, Wentao Li, Yunqian Zhen, Luanluan Jia, Yongzhe Li, Xianjun Pei

**Affiliations:** 1 School of Mechanical Engineering, Southeast University, Nanjing 211189, China; 2 State Key Laboratory of Advanced Welding and Joining, Harbin Institute of Technology, Harbin 150001, China; 3 School of Materials Science and Engineering, Northwestern Polytechnical University, Xi’an 710072, China; 4 Shandong Key Laboratory of Advanced Aluminium Materials and Technology, Weiqiao-UCAS Science and Technology Park, Binzhou 256606, China

**Keywords:** vortex material flow, friction stir welding, weld formation, process parameters, mechanical properties

## Abstract

Vortex- friction stir welding (VFSW) utilizes a stir bar made of an identical material to the workpiece to rub the workpiece’s top surface, which avoids the keyhole defect in conventional friction stir welding. It presents great potential in the repair field of aluminum alloys. In this study, the effect of stir bar diameter, rotation speed, and welding speed on the weld formation was investigated in the VFSW of 6061-T6 aluminum alloy. The weld macrostructure, penetration, and mechanical properties were characterized. The results show that a large diameter of the stir bar can enhance the vortex material flow, increase the heat input, and eliminate the incomplete-penetration defect. The increase in rotation speed within limits can enhance the weld penetration and the mechanical properties of the weld nugget zone (WNZ). However, too high a rotation speed reduces the weld penetration and weakens the mechanical properties of the WNZ. The increase in welding speed reduces the weld penetration but enhances the mechanical properties of the heat affected zone. The incomplete-penetration defect significantly weakens the ductility of the VFSW joint. It can be eliminated by enlarging the stir bar diameter and choosing a moderate rotation speed and a lower welding speed.

## 1. Introduction

Because of their excellent high strength/weight ratio and corrosion resistance, aluminum alloy components are widely used in the aerospace and railway vehicle fields [1,2,3]. During their manufacturing process and the subsequent long-time service, some defects occur inevitably, such as fusion welding defects (porosity and cracks) [4], corrosion pits [5], wear [6], fatigue cracks [7], etc. At the manufacturing stage, once a defect occurs, a repair treatment is necessary, which can greatly save costs [8]. During service, if the above defects occur, the component needs to be replaced. This increases the operational and maintenance costs. If the component with defects can return to service by repair, the lifetime of the component is prolonged [9,10]. Therefore, high-quality repair processes are very necessary for aluminum alloy components.

Some repair processes based on local melting have been developed. For example, Vishnukumar et al. [11] adopted wire arc additive manufacturing to repair the corroded surfaces in AA5052 structures. Yang et al. [12] utilized the cold metal transfer technique to repair the heat-affected zone of the 7075-T651 aluminum alloy MIG welding joint. Li et al. [13] used the identical welding parameters to the original weld to repair the 7N01 aluminum alloy MIG welded joint. However, these repair processes based on local melting always suffer from metallurgical disadvantages such as grain coarsening, alloying element burning loss, and element segregation, which are not good for the mechanical properties of the aluminum alloy components.

Friction stir welding (FSW), as a solid-state welding technique, is a good alternative to avoid the metallurgical disadvantages in local melting repair processes. However, the inherent keyhole defect in FSW needs additional repair treatment. For this purpose, many derivative processes have been developed to repair the keyhole defect. TWI [14] and the NASA Marshall Space Flight Center [15,16] first used friction plug welding to repair the keyhole defect. Huang et al. [17,18] proposed filling friction stir welding to repair the keyhole defect left at the end of the FSW seam. Zhou et al. [19] developed self-refilling friction stir welding to seal the keyhole left by FSW in stainless steel. Ji et al. [20] proposed active-passive filling friction stir repairing to close the keyhole defect in FSW of 7N01-T4 aluminum alloy. Reimann et al. [21] applied refilled friction stir spot welding for termination hole closure in bobbin tool FSW joints in 3 mm thick AA2198-T8 workpieces. Although these methods eliminate the keyhole defect in FSW, the production efficiency is decreased due to the increase in the production procedure.

Recently, Liu et al. [22] proposed a novel modified FSW process, named vortex-material-flow-based friction stir welding, abbreviated as “vortex- friction stir welding” or VFSW. In VFSW, the tool consists of a consumable stir bar which has the identical material to the workpieces and a non-expendable holder which is used to fix and restrain the stir bar. The holder drives the stir bar to rotate at a high speed and rub the workpieces’ top surface. A vortex material flow is formed under the stir bar due to the momentum transfer between the identical materials. With the moving of the tool along the joint line, the vortex material flow drives the material from the front side to the rear side, forming a sound joint [22,23,24,25]. Owing to the above principle of the VFSW process, it avoids the keyhole defect at the weld end. On the contrary, a lug boss is formed at the weld end when the stir bar is separated from the workpiece. This advantage makes the VFSW process show great potential in the aluminum alloy component repair field. To obtain good repair quality, welding process parameters optimization is very important. Previous studies have found that rotation speeds that are too high or too low lead to surface defects due to no vortex material flow occurring [23]. At the same time, too high a welding speed results in an incomplete-penetration defect [23].

Weld penetration is a key factor for repair quality. In VFSW, the weld penetration is highly dependent on the process parameters, such as the stir bar diameter, the rotation speed, and the welding speed. However, the relationship between the weld penetration and the process parameters has not yet been examined. In this study, two stir bars with different diameters were used to reveal the effect of the diameter of the stir bar, the rotation speed, and the welding speed on the weld penetration in VFSW of aluminum alloy. This study provides an important reference for the parameter preference in VFSW of aluminum alloy.

## 2. Experimental Section

### 2.1. Materials

The base material (BM) used in this study is 6061-T6 aluminum alloy. The dimensions of the workpieces are 200 mm in length, 60 mm in width, and 3 mm in thickness. The tensile strength, elongation, and microhardness are 323.5 MPa, 16.7%, and ~100 Hv, respectively [23]. The stir bar is also 6061-T6 aluminum alloy. The holder is made of H13 tool steel.

### 2.2. Process Parameters

**Figure 1:**
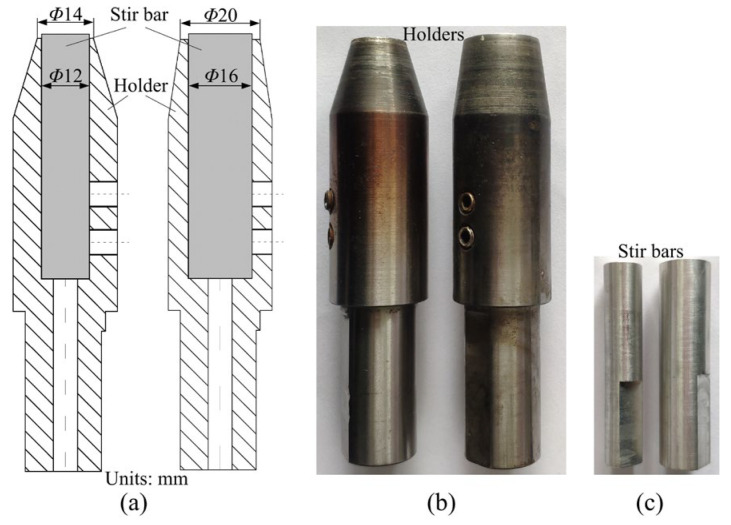
The geometry of the VFSW tool: (**a**) dimensions of the holders; (**b**) the holders used; (**c**) the stir bars before welding.

The process parameters involved in VFSW mainly include the stir bar diameter, rotation speed, and welding speed. As shown in Figure 1, two kinds of stir bars were used in this study. Their diameters (*d*) were 12 mm and 16 mm, respectively. The corresponding diameters of the holder end were 14 mm and 20 mm, respectively. The used rotation speed (*ω*) varied from 600 rpm to 1000 rpm. The welding speed (*v*) ranged from 30 mm/min to 100 mm/min. The above parameters were selected to ensure that good weld surface formation could be obtained during the experiment. The plunge depth of the holder end was 0.3 mm to make sure the holder end was closely touching the workpieces. The type of FSW machine used in this study was HT-JM16×8/1, manufactured by Aerospace Engineering Equipment (Suzhou, China) Co., Ltd.

### 2.3. Welding Procedure

**Figure 2:**
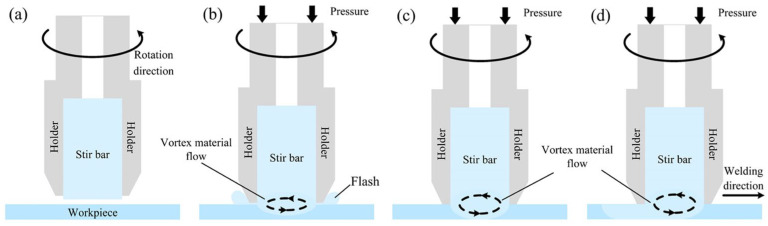
Schematic of the VFSW process procedure: (**a**) tool rotating; (**b**) tool plunging; (**c**) vortex material flow forming; (**d**) tool traversing.

**Figure 3:**
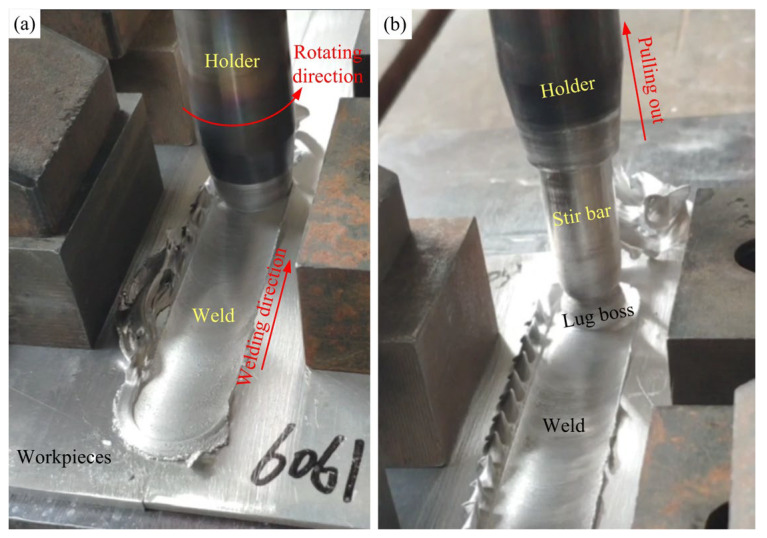
The VFSW process: (**a**) tool traversing stage; (**b**) tool pulling-out stage.

The schematic of the VFSW process is shown in Figure 2. It mainly consists of four steps, i.e., tool rotating, tool plunging, vortex material flow forming, and tool traversing. In the first step, the holder drives the stir bar to rotate at a high speed. Next, the tool is plunged against the workpiece, during which the friction heat is generated and the workpiece material is softened. In the third step, the holder is closely pressed on the workpiece’s top surface. A vortex material flow is formed and becomes stable. Finally, the tool traverses along the welding direction. The region where the vortex material flow passed forms a welding seam. The true VFSW process is shown in Figure 3. During the tool traversing stage, a weld is formed following the holder end. At the tool pulling-out stage, the stir bar is separated from the workpiece, and a lug boss is formed. The region where the vortex material flow passed forms a welding seam. After welding, the macrostructure of the joint was observed using an optical microscope (Leica, DMi8, Wetzlar, Germany) from the transverse cross-section. The local area, which has special features, was characterized by SEM (ZEISS Gemini 500, Oberkochen, Germany). The weld penetration was measured based on the metallograph of the joint using ImageJ software (IJ 1.53k, https://imagej.net/ij/download.html, accessed on 20 June 2022). The tensile properties of the joints under various process parameters were tested using a universal testing machine (DDL100, Sinotest Equipment, Changchun, China). The microhardness was measured by a DHV-1000Z Vickers hardness testing machine (Truer, Shanghai, China) along the mid-line in the thickness direction of the weld.

## 3. Results and Discussion

### 3.1. Weld Morphology

**Figure 4:**
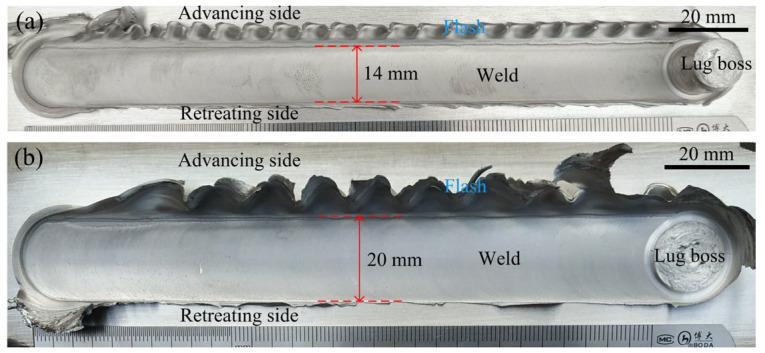
Effect of the stir bar diameter on the weld morphology: (**a**) *d* = 12 mm; (**b**) *d* = 16 mm; (*ω* = 600 rpm, *v* = 30 mm/min).

Figure 4 shows the weld morphology obtained under different stir bar diameters. The widths of the weld’s top are 14 mm and 20 mm, respectively, for the two kinds of stir bars. It means that the width of the weld’s top is determined by the diameter of the holder end but not the diameter of the stir bar. The weld top surface is relatively smooth except for the flashes occurring at the advancing side (AS). The amount of flashes at *d* = 16 mm is more than that at *d* = 12 mm. This is because more material is pushed aside by the holder with a larger diameter. At the end of the weld, a lug boss is formed when the stir bar is separated from the workpieces. The height of the lug boss is different for the two kinds of stir bars. It is higher at *d* = 12 mm than that at *d* = 16 mm. It means that the height of the lug boss is stir-bar-diameter-dependent.

**Figure 5:**
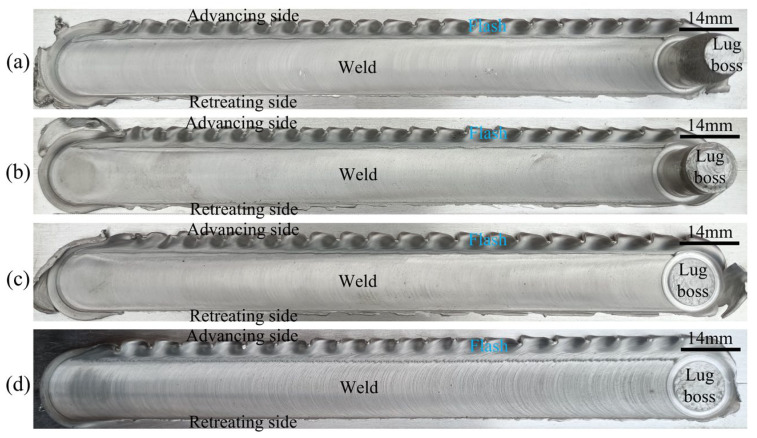
Effect of the rotation speed on the weld morphology: (**a**) *ω* = 700 rpm; (**b**) *ω* = 800 rpm; (**c**) *ω* = 900 rpm; (**d**) *ω* = 1000 rpm; (*v* = 30 mm/min, *d* = 12 mm).

**Figure 6:**
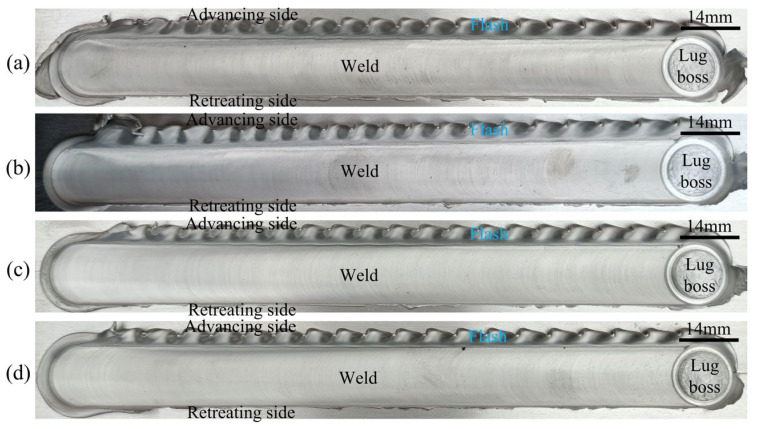
Effect of the welding speed on the weld morphology: (**a**) *v* = 30 mm/min; (**b**) *v* = 50 mm/min; (**c**) *v* = 80 mm/min; (**d**) *v* = 100 mm/min; (*ω* = 900 rpm, *d* = 12 mm).

Figure 5 and Figure 6 show the effects of the rotation speed and the welding speed on the weld morphologies, respectively. The height of the lug boss is also rotation-speed-dependent but not welding-speed-dependent. This is because the rotation speed affects the heat softening and the deformation hardening of the junction between the stir bar and the workpiece simultaneously, which has been discussed in detail in a previous study [25]. The stir bar diameter and the rotation speed jointly determine the weakest zone between the vortex material flow and the holding end of the stir bar during the holder being pulled out. The existence of the lug boss replaced the keyhole defect inherently occurring in conventional FSW. This is a great advantage for the VFSW process. In addition, the rotation speed also affects the weld surface’s smoothness. As the rotation speed increases to 1000 rpm, the weld top surface is significantly roughened, as shown in Figure 5d. Meanwhile, the welding speed nearly does not affect the weld surface smoothness. This is attributed to the obvious change in welding temperature caused by the rotation speed [24], while the small change is caused by the welding speed.

### 3.2. Weld Macrostructure

**Figure 7:**
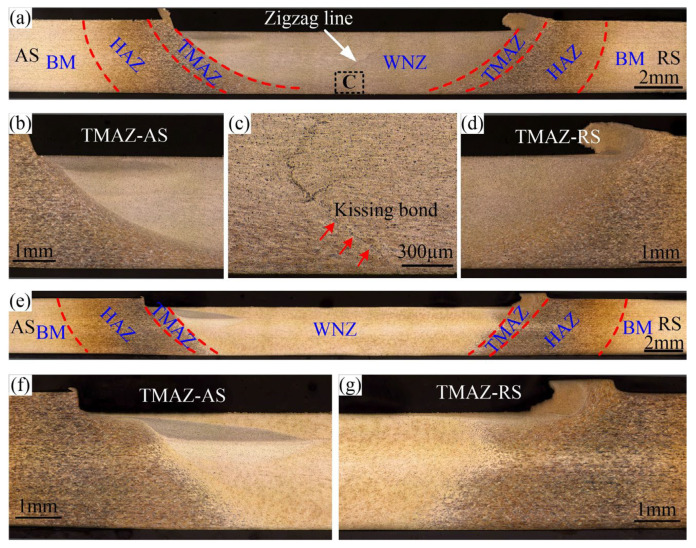
Characteristics of the weld transverse cross-section: (**a**) *d* = 12 mm; (**b**–**d**) the corresponding magnified pictures of TMAZ-AS, featured region C, and TMAZ-RS in (**a**); (**e**) *d* = 16 mm; (**f**,**g**) the corresponding magnified pictures of TMAZ-AS and TMAZ-RS in (**e**); (*ω* = 600 rpm, *v* = 30 mm/min).

Figure 7 shows the weld macrostructures obtained under different stir bar diameters. The weld macrostructure in VFSW is like that in conventional FSW. From the center to two sides, it contains the weld nugget zone (WNZ), the thermal/mechanical-affected zone (TMAZ), the heat-affected zone (HAZ), and the base material (BM). The definition of the advancing side (AS) and the retreating side (RS) are also appropriate for the VFSW process. The diameter of the stir bar has a decisive influence on the shape of the WNZ, which can be verified by the width of the weld’s bottom. For example, at the same rotation speed and welding speed, the weld’s bottom at *d* = 16 mm is significantly wider than that at *d* = 12 mm, as shown in Figure 7a,e. The shapes of TMAZ are also different for different stir bar diameters, as shown in Figure 7b,d,f,g. The boundary between the TMAZ and the WNZ is more obvious for a small stir bar diameter. This indicates that the change in grain size is more significant at *d* = 12 mm. The area of the HAZ at *d* = 16 mm is also significantly larger than that at *d* = 12 mm. These mean that the larger diameter of the stir bar elevates the heat input. The larger diameter of the stir bar also enhances the material flow at the weld’s bottom, as shown in Figure 7e. In addition, a zigzag line exists in the WNZ at *d* = 12 mm, but no obvious zigzag line occurs at *d* = 16 mm. The zigzag line is believed to be the remnants of the oxide layer on the butt surface, which is broken into oxide particles during the welding [26,27,28]. This means that the larger diameter of the stir bar can eliminate the zigzag line via enhanced plastic deformation. At the lower part of the WNZ, at *d* = 12 mm, a kissing bond defect is observed, as shown in Figure 7c.

**Figure 8:**
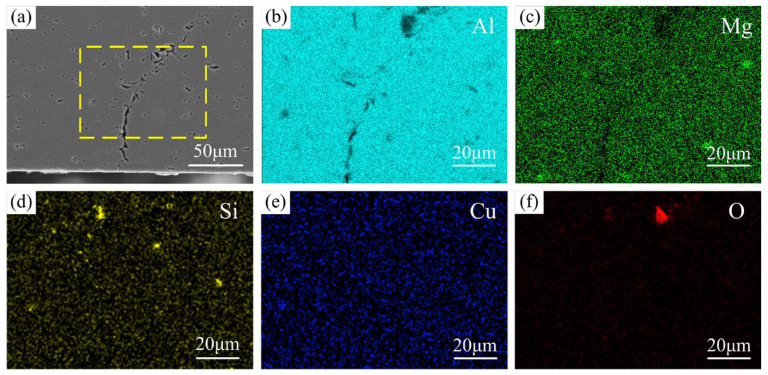
EDS mapping of a typical incomplete-penetration defect (obtained at *d* = 12 mm, *ω* = 600 rpm, and *v* = 30 mm/min): (**a**) SEM image; (**b**–**f**) EDS images showing the elemental distributions of Al, Mg, Si, Cu, and O in the selected area by the yellow dotted line in (**a**).

Figure 8 shows the EDS mapping of a typical kissing bond defect. The Al and Mg elements are missing within the defect. This proved that the kissing bond defect contains cracks. The aggregation of the O element is also observed. It indicates that oxides also exist in the kissing bond defect. In a word, the kissing bond defect in VFSW essentially consists of discontinuous cracks and oxides, which are bound to seriously affect the mechanical properties of the joint. Therefore, this kind of defect is defined as an incomplete-penetration defect in this study.

### 3.3. Weld Penetration

**Figure 9:**
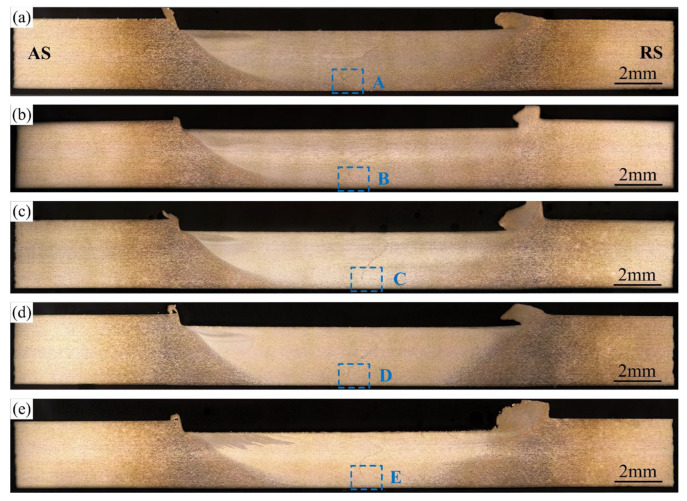
Weld transverse cross-sections to various rotation speeds: (**a**) *ω* = 600 rpm; (**b**) *ω* = 700 rpm; (**c**) *ω* = 800 rpm; (**d**) *ω* = 900 rpm; (**e**) *ω* = 1000 rpm; (*v* = 30 mm/min, *d* = 12 mm).

**Figure 10:**
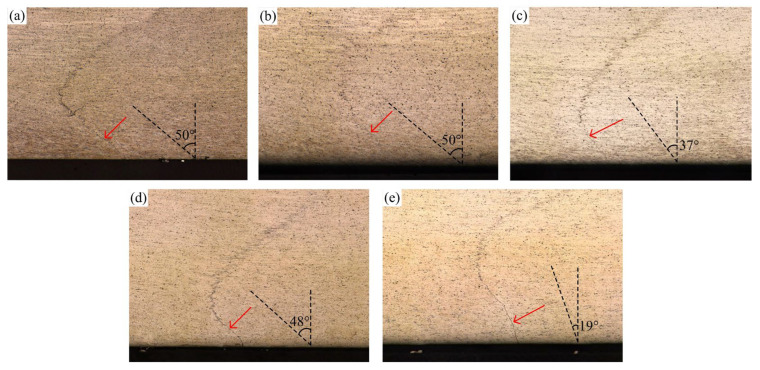
Magnified incomplete-penetration defects: (**a**–**e**) corresponding to the featured region A–E in Figure 9.

Figure 9 shows the weld transverse cross-sections at various rotation speeds. The shape of the stir zone has no obvious change as the rotation speed increases. The incomplete-penetration defects exist under all conditions. To observe the incomplete-penetration defects, the magnified figures of the local area marked by A–E in Figure 9 are shown in Figure 10. The original butting surface has been distorted in all the incomplete-penetration defects. However, the interface is relatively smooth near the weld bottom surface. It gradually becomes jagged far away from the bottom surface, which means that the bonding quality is improved. The distortion degree of the original butting surface can be described by the tilted angle of the interface. As shown in Figure 10, at 600 and 700 rpm, the tilted angle of the interface reaches 50°, which means the vortex is strong. As the rotation speed increases, the tilted angle of the interface decreases. Especially at 1000 rpm, the tilted angle is only 19°, which means that the vortex becomes very weak if the rotation speed is too high.

**Figure 11:**
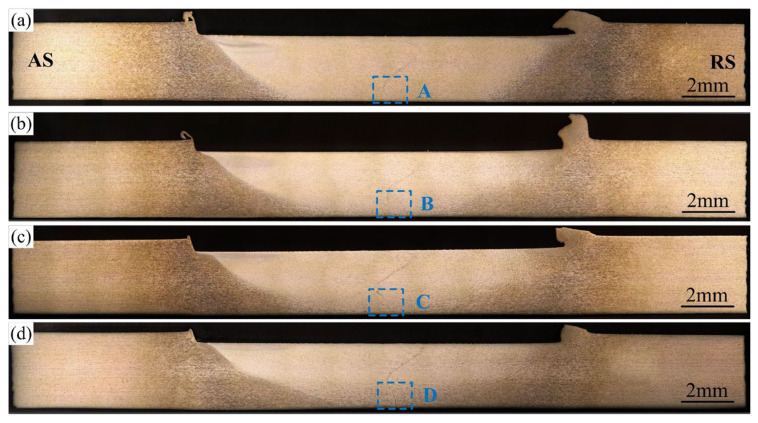
Weld transverse cross-sections to various welding speeds: (**a**) *v* = 30 mm/min; (**b**) *v* = 50 mm/min; (**c**) *v* = 80 mm/min; (**d**) *v* = 100 mm/min; (*ω* = 900 rpm, *d* = 12 mm).

**Figure 12:**
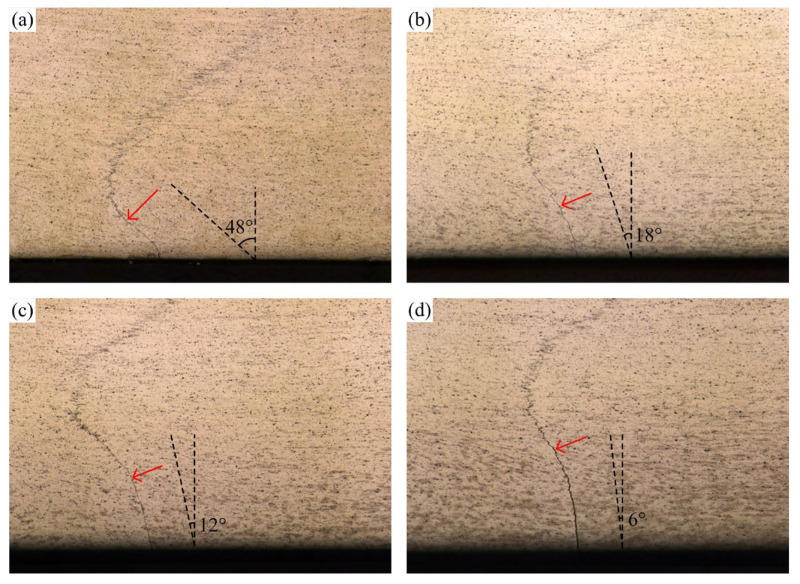
Magnified incomplete-penetration defects: (**a**–**d**) corresponding to the featured region A–D in Figure 11.

Figure 11 shows the weld transverse cross-sections at various welding speeds. Different from those at various rotation speeds, the volume of the stir zone becomes smaller and smaller as the welding speed increases. The magnified incomplete-penetration defects in Figure 12 show that the distortion of the original interface becomes weaker and weaker as the welding speed increases. The tilted angle of the original butting surface decreases from 48° to 6° as the welding speed increases from 30 mm/min to 100 mm/min. This means that the welding speed has a great influence on the intensity of the vortex material flow beneath the stir bar.

**Figure 13:**
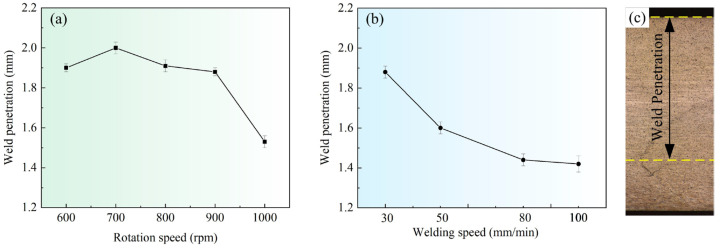
Weld penetration: (**a**) variation with rotation speeds, *v* = 30 mm/min; (**b**) variation with welding speeds, *ω* = 900 rpm; (**c**) schematic of the weld penetration measurement; (*d* = 12 mm).

Figure 13 shows the weld penetration to the rotation speed and the welding speed. The weld penetration was measured based on the metallographs, as shown in Figure 13c. The weld penetration is equal to subtracting the thickness of the incomplete-penetration defect from the total weld thickness. Owing to the existence of the incomplete-penetration defect at *d* = 12 mm, the weld penetration is always smaller than the thickness of the workpieces. This allows us to detect the relationship between the weld penetration and the process parameters. In Figure 13a, as the rotation speed increases, the weld penetration first slightly increases to ~2 mm at *ω* = 700 rpm, and then significantly decreases to ~1.53 mm at *ω* = 1000 rpm. As the welding speed increases from 30 mm/min to 100 mm/min, the weld penetration first rapidly decreases and then slowly decreases. The factors determining the welding penetration mainly include the depth and the intensity of the vortex material flow in VFSW. The former determines the volume of plastic deformation. The latter affects the metallurgical bonding quality. Owing to the boundary conditions’ constraint for the vortex material flow, the intensity of the vortex material flow at the weld lower part is quite weak under some parametric conditions, although plastic deformation occurs. Therefore, the incomplete penetration is formed. According to the weld penetration, it can be concluded that increasing the rotation speed can enhance the vortex to some extent. It is easily understandable that the high rotation speed increases the momentum input into the vortex. However, too high a rotation speed cannot enhance the intensity of the vortex. This is because the resulting high temperature at too high a rotation speed severely softens the material. Accordingly, the viscosity of the material declines. Thus, the efficiency of the momentum transfer decreases significantly, resulting in the weakening of the vortex intensity at the weld lower part.

The welding speed affects the vortex from two aspects. On the one hand, increasing welding speed reduces the heat input per unit distance, resulting in the weakening of the material softening. On the other hand, the kinematics and the dynamics of the vortex are superimposed by the welding speed. Intuitively, the high welding speed is adverse to the vortex, but it needs further investigation. To sum up, both the depth and the intensity of the vortex decrease as the welding speed increases. Therefore, the welding penetration decreases gradually as the welding speed increases.

### 3.4. Mechanical Properties

**Figure 14:**
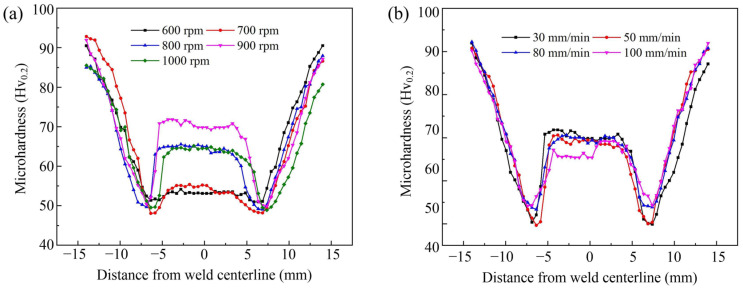
Microhardness distribution on the transverse cross-section of the joint: (**a**) variation with rotation speeds, *v* = 30 mm/min; (**b**) variation with welding speeds, *ω* = 900 rpm; (*d* = 12 mm).

**Figure 15:**
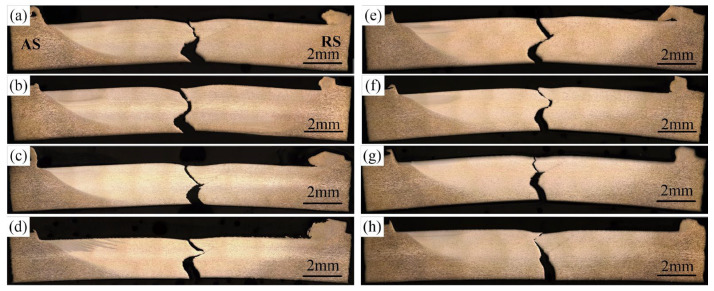
Fracture locations at various rotation speeds and welding speeds: (**a**–**e**) *v* = 30 mm/min; (**f**–**h**) *ω* = 900 rpm; (**a**) *ω* = 600 rpm; (**b**) *ω* = 700 rpm; (**c**) *ω* = 800 rpm; (**d**) *ω* = 900 rpm; (**e**) *ω* = 1000 rpm; (**f**) *v* = 50 mm/min; (**g**) *v* = 80 mm/min; (**h**) *v* = 100 mm/min; (*d* = 12 mm).

**Figure 16:**
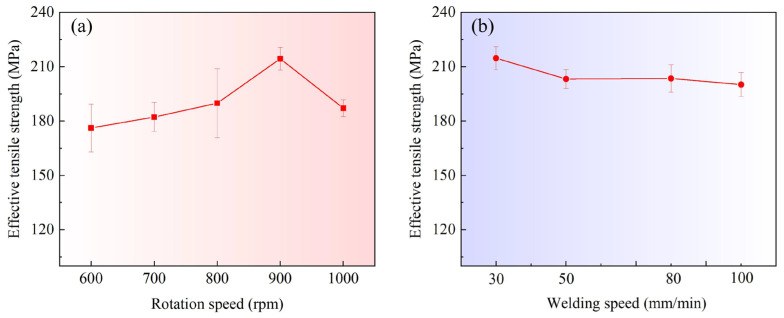
Relationship between the effective tensile strength and the welding parameters: (**a**) weld penetration to rotation speed; (**b**) weld penetration to welding speed; (*d* = 12 mm).

Figure 14 shows the microhardness profile of the joints measured along the horizontal mid-line of the weld. Figure 15 shows the fracture locations of the joints during the uniaxial tensile tests. Figure 16 shows the corresponding effective tensile strength. All the data were obtained at *d* = 12 mm. The effective tensile strength in this study is defined as the tensile load divided by the weld penetration. Owing to the existence of incomplete-penetration defects, the original tensile strength cannot characterize the bonding quality within the weld penetrated zone. Therefore, effective tensile strength is introduced in this study. It is reasonable because the incomplete-penetration defect mainly consists of discontinuous cracks and oxides, which nearly have no bearing capacity. What needs to be pointed out is that the effective tensile strength only can be used to evaluate the WNZ where metallurgical bonding occurs. It cannot be used to evaluate the whole joint because all the joints were fractured at the WNZ during the tensile test due to the existence of the incomplete-penetration defect, as shown in Figure 15.

The measured mechanical properties for when the welding speed is constant and 30 mm/min are shown in Figure 14a and Figure 16a. The rotation speed mainly affects the microhardness in the WNZ. With the increase in the rotation speed, the average microhardness in the WNZ first increases and then decreases. The effective tensile strength in Figure 16a also presents the same change trend. At *ω* = 900 rpm, the microhardness has a maximum value of up to ~70 Hv. The corresponding effective strength reaches ~215 MPa, which is equivalent to that in conventional FSW [29]. Liu et al. [30] reported that the strengthening mechanism of the WNZ in the VFSW of 6061-T6 aluminum alloy is mainly solid solution strengthening. A higher temperature promotes solid solution strengthening [31]. Therefore, the high temperature caused by a relatively high rotation speed is beneficial to the tensile strength of the WNZ. This can be used to explain why the microhardness increases with the increasing rotation speed. However, if the rotation speed is too high, the interface material between the stir bar and the workpieces is severely softened and even locally liquefied. The momentum transfer from the stir bar to the workpieces is therefore suppressed. This not only decreases the intensity of the vortex but also reduces the welding temperature. Therefore, both the microhardness and the effective tensile strength at *ω* = 1000 rpm decrease. The HAZ has the minimum microhardness for the joint. The minimum value at various rotation speeds changes little. This is because the over-aging effect occurs in the HAZ [30]. The precipitates in the HAZ were mainly the β′ phase, which evolved from the β″ phase in the BM and coarsened under the heating, leading to the severe softening of the HAZ. The heating time is the determining factor for over-aging [31]. Therefore, at the same welding speed, the HAZ material close to the TMAZ nearly undergoes the same over-aging treatment, and the minimum microhardness in the HAZ hardly changes.

The microhardness and the effective tensile strength are shown in Figure 14b and Figure 16b, respectively, for when the rotation speed is constant and 900 rpm. The welding speed ranges from 30 mm/min to 100 mm/min. The welding speed mainly affects the microhardness of the HAZ, as shown in Figure 14b. As the welding speed increases, the minimum microhardness value in the HAZ also increases. This is because the welding speed determines the heating and cooling rates. The dwell time of high temperatures at higher welding speeds is shorter. Therefore, the over-aging occurring at higher welding speeds is weaker. In the WNZ, a higher welding speed results in a shorter time for solution treatment. However, owing to the solid solution being very fast [31], the effect of the welding speed on the mechanical properties of the WNZ is very small. The effective tensile strength presents very small fluctuations with the increase in welding speed, as shown in Figure 16b. At *v* = 100 mm/min, because the weld penetration is only 1.42 mm, the microhardness measured positions may enter the incomplete-penetration defect zone. Therefore, an obvious decrease in the microhardness is observed, as shown in Figure 14b.

**Figure 17:**
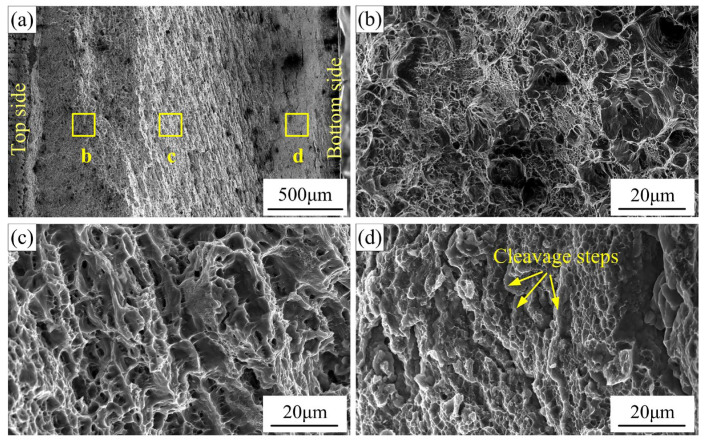
Typical fracture surface morphology of the joint: (**a**) macro-morphology; (**b**–**d**) magnified morphologies of the areas b, c, and d in (**a**); (*ω* = 900 rpm, *v* = 30 mm/min, *d* = 12 mm).

Figure 17 shows a typical fracture surface morphology of the joint at the rotation speed of 900 rpm and the welding speed of 30 mm/min. The local magnified figures of the upper part, the mid part, and the lower part are shown in Figure 17b–d, respectively. In Figure 17b, a lot of large and deep dimples are observed, which are the typical characteristics of ductile fracture, indicating the good bonding quality at the weld’s upper part. In Figure 17c, the dimples are relatively small and shallow, which indicates that relatively small plastic deformation occurred at the weld mid-part during the tensile test, showing the decline of the ductility. In Figure 17d, the dimples are very small and even nearly disappeared. No obvious tear ridge exists. Some cleavage steps are even observed. These results show that the joint fracture is in a mixed ductile/brittle fracture mode. This indicates that the crack propagation originated from the incomplete-penetration defect and traverses through the good weld part during the tensile tests.

## 4. Conclusions

The effects of stir bar diameter, rotation speed, and welding speed on the weld penetration and the joint quality in VFSW of 6061-T6 aluminum alloy have been investigated carefully. The following conclusions can be drawn:

(1) A larger diameter of the stir bar can enhance the vortex material flow in VFSW, increase the heat input, and eliminate the incomplete-penetration defect. For a sheet of 3 mm in thickness, a stir bar of 16 mm in diameter is suitable to obtain a defect-free weld.
(2) Owing to the resulting high temperature and large momentum input, the increase in rotation speed within limits can enhance the weld penetration and the mechanical properties of the WNZ. However, too high a rotation speed reduces the weld penetration and weakens the mechanical properties of the WNZ. With *d* = 12 mm and *v* = 30 mm/min, the maximum weld penetration reaches ~2 mm at *ω* = 700 rpm, and the maximum effective tensile strength reaches ~215 MPa at *ω* = 900 rpm.
(3) Because of the decrease in the heat input per unit distance, the increase in welding speed reduces the weld penetration invariably but enhances the mechanical properties of the HAZ due to the over-aging becoming weak. However, it nearly does not affect the mechanical properties of the WNZ.
(4) As a crack origin, the incomplete-penetration defect significantly weakens the ductility of the VFSW joint.

## Data Availability

All data generated or analyzed during this study are included in this published article.
